# High-performance modified LDH for green one-pot synthesis of pyrido[2,3-*d*]pyrimidines

**DOI:** 10.1016/j.heliyon.2024.e41149

**Published:** 2024-12-12

**Authors:** Sarieh Momeni, Ramin Ghorbani-Vaghei

**Affiliations:** a Department of Organic Chemistry, Faculty of Chemistry and Petroleum Sciences, Bu-Ali Sina University, Hamedan, Iran; b Department of Organic Chemistry, Faculty of Chemistry, Guilan University, Rasht, Iran

**Keywords:** Layered double hydroxides, LDH, Pyrido[2,3-*d*]pyrimidine derivatives, MCRs, Nano catalyst

## Abstract

This study introduces a new nano catalyst tailored for the eco-friendly synthesis of pyrido[2,3-*d*]pyrimidine via a three-component one-pot reaction involving benzaldehydes, malononitrile, and uracil. To achieve this objective, we anchored copper acetate onto the surface of layered double hydroxides modified with 1,3‑benzenedisulfonyl amide (BDSA) (LDH@PTRMS@BDSA@Cu(NO_3_)_2_), which exhibited remarkable activity and selectivity. The main benefits of this method include high product yield, swift reaction times, straightforward purification, catalyst reusability, and the employment of a mild reaction process. Furthermore, the catalyst maintained its effectiveness even after being recycled four times without notable degradation in catalytic performance.

## Highlights

• Development of a new nanocatalyst incorporating a layered double hydroxides coated with 1,3-benzenedisulfonamide.
• The catalyst showed exceptional efficiency in the synthesis of pyrido[2,3-d]pyrimidines.
• Products were obtained under green conditions, utilizing a one-pot three-component reaction.
• Advantages include high product yield, short reaction duration, and low operating temperature.
• Catalyst successfully recycled four times with negligible loss of efficiency per cycle.

## Graphical abstract

**Figure:**
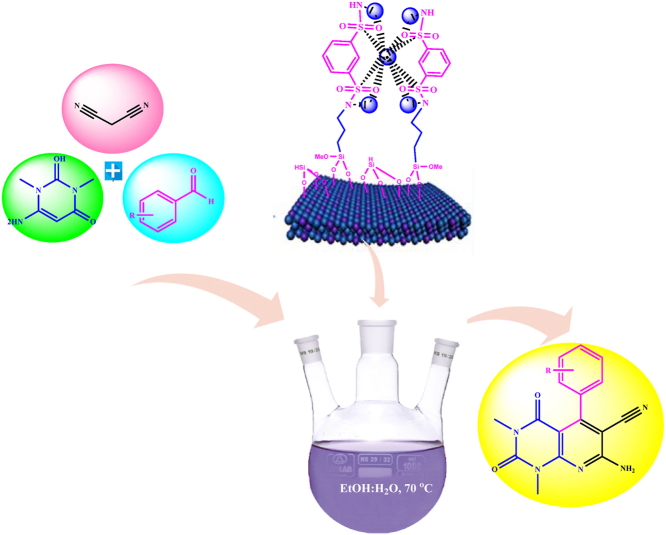

## 1 Introduction

Lately, the synthesis of layered nanohybrids using inorganic nanomaterials has become pivotal in catalysts. Layered double hydroxides (LDH) stand out as a prevalent choice among these inorganic matrices [[1], [2], [3]]. The basic structure consists of layers with a positive charge, similar to brucite, which are made up of a mixture of metal hydroxides. Within these layers, there exists a space between them that contains anions and water molecules to balance the charge. Metal cations are located in the centers of interconnected octahedra, while hydroxide ions at the vertices combine to form continuous two-dimensional sheets [4,5].

The general formula for LDH is expressed as [M^2+^_(1-x)_ M_x_^3+^(OH)_2_](A^n−^) _x/n_.zH_2_O]. Here, M(II) represents a divalent metal cation such as Mg, Mn, Ni, Zn, Co, Fe, or Cu, while M(III) denotes a trivalent metal cation like Al, Fe, Co, Ni, Mn, or Cr. An^−^ represents an interlayer anion like Cl^−^, F^−^, CO_3_^2−^, NO_3_^−^, or SO_4_^2−^. The parameter “x" represents the layer charge, usually falling within the range of 0.2–0.33, correlating with the molar proportion of M(II) to the total of M(II) and M(III). LDHs have attracted growing attention as carriers for cellular delivery, owing to their inert nature and compatibility with biological systems [[6], [7], [8], [9]].

The burgeoning field of chemistry is advancing novel methodologies with a focus on environmental considerations. Chemists are exploring eco-friendly techniques such as the use of benign solvents (e.g., water), solvent-free syntheses, cost-effective catalysts, one-pot multicomponent reactions, and more [[10], [11], [12]]. Nanocomposites play a pivotal role in green synthesis, capitalizing on their nano-scale dimensions to leverage nanoparticles as catalysts. The catalytic efficacy is intricately tied to the surface area of the nanoparticles, with smaller particle sizes yielding larger surface areas, thus maximizing catalytic efficiency with minimal catalyst usage. Furthermore, in nanocomposites, selectivity aids in eliminating unwanted byproducts. Notably, nanocomposites exhibit heterogeneity alongside high catalytic activity, facilitating their easy separation from reaction mixtures upon completion [[13], [14], [15]].

Multicomponent reactions (MCRs) have long been a cornerstone of organic synthesis due to their wide-ranging applications, particularly in pharmaceutical research. As a result, there has been a notable surge in interest towards producing a wide array of intricate compounds with substantial yields, alongside the development of streamlined purification methods, within the realm of combinatorial chemistry [16,17]. Recent years have witnessed remarkable advancements in MCRs, with substantial efforts directed towards the development of novel catalytic MCRs. The protocol offers notable advantages, including high yields, short reaction times, enhanced atom economy, and reductions in solvent usage and energy consumption [[18], [19], [20], [21]].

Uracil and its fused derivatives serve as vital biological reagent, facilitating the synthesis of anticancer and antiviral medications. There has been a surge of interest in pyrido[2,3-*d*]pyrimidines, which are fused uracil derivatives, due to their wide range of biological and pharmacological effects. These effects encompass various roles, such as serving as antitumor agents [22] and antihypertensives [23], hepatoprotectives [24], cardiotonic [25], anticonvulsant [26], and antibacterial [27]. The functionalization of uracil derivatives has facilitated the preparation of a variety of compounds, including pyramido-pyrimidines [28], pyrazolo-pyridines [29], pyrazolopyrimidines [30], pyrido-purines [31], and xanthine [32] derivatives.

Numerous studies focus on the synthesis of complex molecules involving the manipulation of uracil derivatives, including palladium-catalyzed oxidative coupling [33], nanocrystalline MgO in water [34], glycerol [35], Al-HMS-20 in EtOH [36], L-proline [37], sulfonic acid-functionalized SBA-15 [38], 1,4-diazabicyclo[2.2.2]octane (DABCO)-functionalized ionic liquid [39], diammonium hydrogen phosphate (DAHP) [40], SBA-Pr-SO_3_H [20], Fe_3_O_4_ nanoparticles in EtOH [41], and tetra-n-butyl ammonium bromide (TBAB) [42].

It's evident that these methods often entail unfavorable conditions, such as the use of organic solvents, extended reaction durations, and low yields. Hence, there is a pressing need to devise operational methods for synthesizing these molecules.

In our investigation, we fabricate a novel catalyst utilizing a layered double hydroxide functionalized with the 1,3-benzenedisulfonyl amide ligand, and further stabilized with copper acetate metal (LDH@PTRMS@BDSA@Cu(NO_3_)_2_). Subsequently, we demonstrate a three-component, one-pot synthesis method for pyrido[2,3-*d*]pyrimidine derivatives, achieved through the reaction of benzaldehyde, malononitrile, and 6-amino-1,3-dimethyluracil in the presence of this catalyst. Notably, the derivatives are synthesized with high yield (87–94 %), minimal reaction time (13–20 min), and under environmentally friendly conditions. Additionally, the catalyst demonstrated impressive reusability, as it was successfully recycled and reused for up to four consecutive reaction cycles without significant reduction in its catalytic efficiency.

## 2 Experimental

### 2.1 Protocol for synthesizing layered double hydroxides (LDHs)

Zn/Cr-LDH synthesis was conducted according to the previously outlined method, summarized as follows: Initially, salts of Zn (NO_3_)_2_·6H_2_O and Cr (NO_3_)_3_·9H_2_O, in a 2:1 M ratio, were dissolved in deionized water. The solution's pH was then adjusted to 11.5 using a 2 M NaOH aqueous solution under vigorous stirring and maintained at the same temperature for 18 h. The resultant turquoise compound was filtered, washed with distilled water, and subsequently dried in an oven at 60 °C for 24 h [43].

### 2.2 Method for synthesizing LDHs coated with 3-chloropropyltrimethoxysilane (LDH@PTRMS)

3-Chloropropyltrimethoxysilane was used to activate LDH. First, 1 g of the synthesized LDH was added to 50 mL of toluene and dispersed in a sonication bath for 20 min at room temperature. Following this, 2 mL of 3-chloropropyltrimethoxysilane was added to the solution, which was refluxed with continuous stirring over a 24-h period. After refluxing, the formation of a precipitate was observed, which we collected using filter paper. Subsequently, the precipitate underwent several washes with toluene and ethanol to remove impurities. Finally, the washed material was dried in an oven at 60 °C to ensure complete removal of solvents and moisture [43].

### 2.3 Method for synthesizing ligand of 1,3-benzenedisulfonyl amide

Initially, 16.5 mmol of phosphorus pentachloride was introduced, serving as the chlorinating reagent, into a vessel containing 5.00 g (18 mmol) of 1,3-benzenedisulfonic acid disodium salt. The mixture was then heated to 65 °C, allowing the reaction to progress for 2 h. Upon completion, a solution comprising ice (150 g) and chloroform (150 mL) was added, facilitating the partitioning of the organic layer containing 1,3-benzenedisulfonyl chloride. Subsequent to this separation, in a 50 mL flask, we combined 1 g of 1,3-benzenedisulfonyl chloride with 5 mL of 30% ammonia solution, and the resulting mixture was refluxed for 12 h. Upon conclusion of the reaction, the flask was sealed with paraffin and cooled to 0 °C to initiate crystal formation. The resultant crystals were then collected and subjected to drying procedures [19].

### 2.4 Method for synthesizing LDH@PTRMS@BDSA

The layered double hydroxides, having been treated with 3-chlorotrimethoxysilane (0.3 g), was dispersed in 80 mL of toluene via ultrasonication for a duration of 15 min. Subsequently, 0.55 g of BDSA was introduced into the dispersion, initiating a reflux process lasting 24 h. After completion, the resulting mixture underwent filtration, followed by three successive washes with distilled water and ethanol. Finally, the product was dried in an oven at 60 °C for a period of 24 h to remove any residual solvent and moisture [19].

### 2.5 Method for synthesizing LDH@PTRMS@BDSA@Cu(NO_3_)_2_

**Scheme 1:**
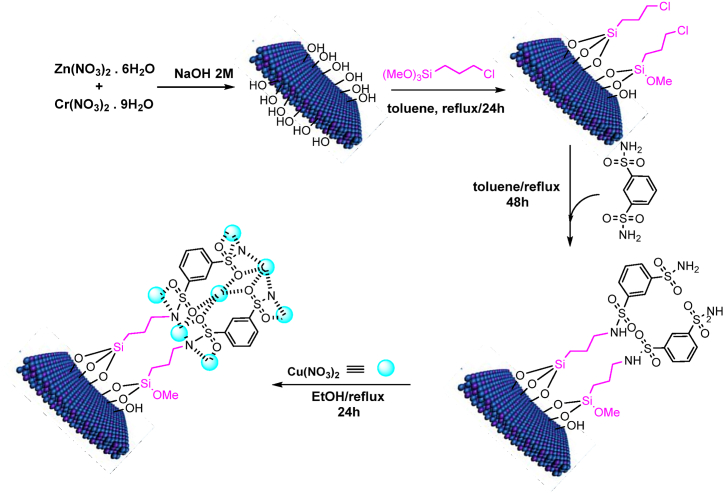
Synthesis steps of LDH@PTRMS@BDSA@Cu(NO_3_)_2_ catalyst.

To load Cu onto the LDH@PTRMS@BDSA, 0.3 g of the metal was placed into a flask containing LDH@PTRMS@BDSA dispersed in 20 mL of ethanol (totaling 0.5 g). After dispersing for 5 min, the mixture was refluxed for 12 h. Subsequently, the LDH@PTRMS@BDSA@Cu(NO_3_)_2_ nanoparticles were separated by centrifugation, followed by washing with ethanol to remove impurities. Finally, the washed nanoparticles were dried under vacuum conditions at 60 °C for a period of 24 h to ensure complete removal of moisture (Scheme 1).

### 2.6 Method for synthesizing pyrido[2,3-*d*]pyrimidine derivatives in the presence of LDH@PTRMS@BDSA@Cu(NO_3_)_2_ nanoparticles

In a test tube, a mixture of 6-amino-1,3-dimethylpyrimidine-2,4(1*H*,3*H*)-dione (1 mmol), malononitrile (1 mmol), aromatic aldehyde derivatives (1 mmol), and nanoparticles LDH@PTRMS@BDSA@Cu(NO_3_)_2_ (0.05 g) was stirred in a solution of water and ethanol (0.5:0.5 mL) at 70 °C using a magnetic stirrer for an appropriate duration. The reaction progress was monitored by TLC (using ethyl acetate/normal hexane: 4.8). After the reaction concluded and the desired compound formed, the mixture was allowed to cool down to ambient temperature. To isolate the catalyst, 3 mL of hot ethanol or chloroform was added to the reaction mixture and stirred for 2 min, the catalyst remained insoluble while the reaction mixture dissolved. The centrifugation process was used to isolate the catalyst (LDH@PTRMS@BDSA@Cu(NO_3_)_2_), which was subsequently washed and dried in an oven maintained at 60 °C. Subsequently, the solvent from the reaction mixture was evaporated, and the products were dissolved in ethanol via nebulization, resulting in the synthesis of compounds with high yield. Product identification was conducted using FTIR, ^1^H NMR, and ^13^C NMR spectra, and the melting points of all products were recorded.

## 3 Results and discussion

### 3.1 Examining the spectroscopic profile of the catalyst: copper nitrate anchored onto the layered double hydroxides and coated with BDSA

**Fig. 1:**
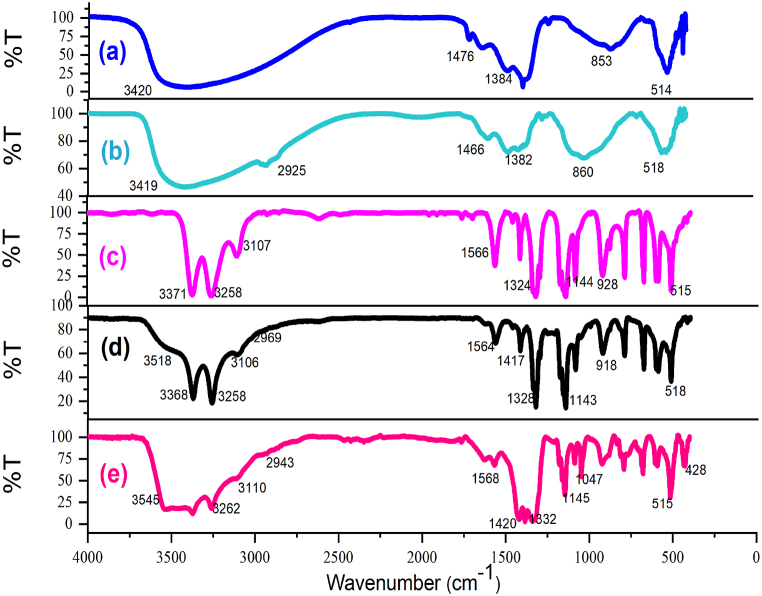
FTIR spectra of (a) LDH, (b) LDH@PTRMS, (c) 1,3-benzenedisulfonylamide (BDSA) ligand, (d) LDH@PTRMS@BDSA and (e) LDH@PTRMS@BDSA@Cu(NO_3_)_2_

Following the synthesis of the catalyst, a range of analyses, including Fourier-transform infrared spectroscopy (FT-IR), field emission scanning electron microscopy (FESEM), X-ray diffraction (XRD), energy dispersive X-ray spectroscopy (EDX), and thermal decomposition analysis (DSC, TGA), were conducted to confirm and identify the LDH@PTRMS@BDSA@Cu(NO_3_)_2_ catalyst. Fig. 1 illustrates the FT-IR spectrum of various components: (a) the double hydroxide layer, (b) LDH@PTRMS, (c) 1,3-benzenedisulfonyl amide (BDSA) ligand, (d) LDH@PTRMS@BDSA, and (e) LDH@PTRMS@BDSA@Cu(NO_3_)_2_. Part (a) corresponds to LDH, where peaks ranging from 2500 to 3420 cm^−1^are attributed to the hydroxides on LDH, while those in the region of 1384–1476 cm^−1^ denote the stretching vibrations of the interlayer nitrate anion. Additionally, the peak at 853 cm^−1^ is associated with metal-oxygen stretching vibrations. Part B represents LDH functionalized with 3-chloropropyltrimethoxysilane, with the peak at 2925 cm^−1^ indicating C-H stretching vibrations [43]. Part (c) pertains to the BDSA ligand, where peaks at 3371 and 3258 cm^−1^ signify NH stretching vibrations, and stretching vibrations of sulfonyl groups are observed in the 1324 and 1144 cm^−1^ regions. Part (d) corresponds to the attachment of the ligand on LDH@PTRMS, where stretching vibrations of the LDH hydroxyl groups are evident at 3518 cm^−1^, peaks related to the amine ligand are observed at 3368 and 3258 cm^−1^, and sulfonyl groups also appear at 1328 and 1143 cm^−1^ [19]. Finally, Part (e), representing the final stage of catalyst synthesis, demonstrates the incorporation of copper nitrate onto the double hydroxide layer coated with the 1,3-benzenedisulfonyl amide ligand. Observations indicate shifts in NH ligand 1,3-benzenedisulfonyl amide peaks and a reduction in the intensity of S=O peaks, suggesting their interaction with the nanomaterials. Notably, peaks at 515, 575, and 1385 cm^−1^ correspond to Cu(NO_3_)_2_, confirming its successful immobilization.

**Fig. 2:**
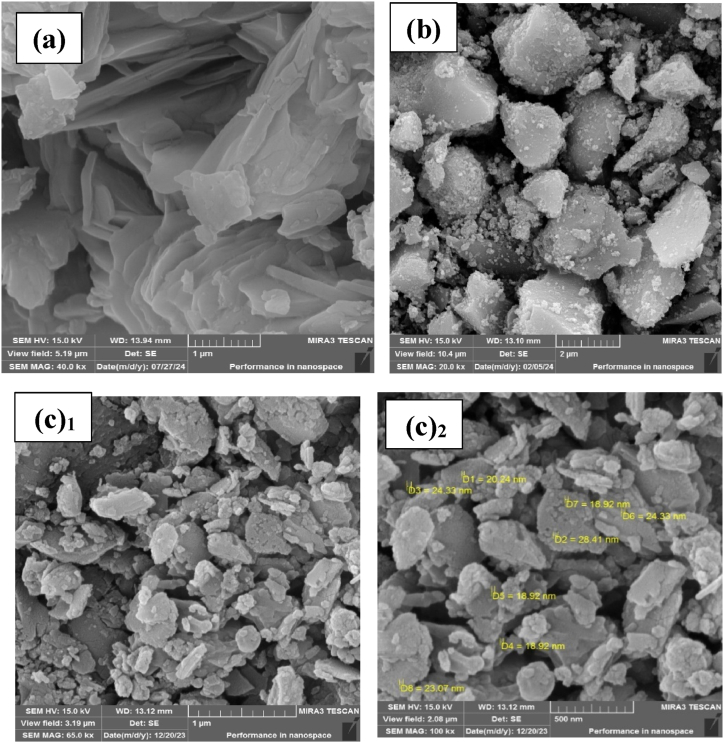
FESEM images of (a) LDH, (b) LDH@PTRMS and (c_1_-c_2_) LDH@PTRMS@BDSA@Cu(NO_3_)_2_ catalyst.

Scanning electron microscope (SEM) images provide valuable insights into particle morphology and size. Fig. 2 depicts SEM images of LDH, LDH@PTRMS and the LDH@PTRMS@BDSA@Cu(NO_3_)_2_ nano-catalyst, in part a, the LDH are depicted, with the plates stacked on top of each other, each approximately 2 μm in size. Part b illustrates the LDH@PTRMS composite, clearly demonstrating the functionalization of LDH with TRMS, where silica particles are positioned on the surface. Part c presents the LDH@PTRMS@BDSA@Cu(NO_3_)_2_ nano-catalyst, showing copper nanoparticles uniformly distributed across the sheet structures, creating a roughened surface. Furthermore, the images indicate particle sizes around 20 nm.

**Fig. 3:**
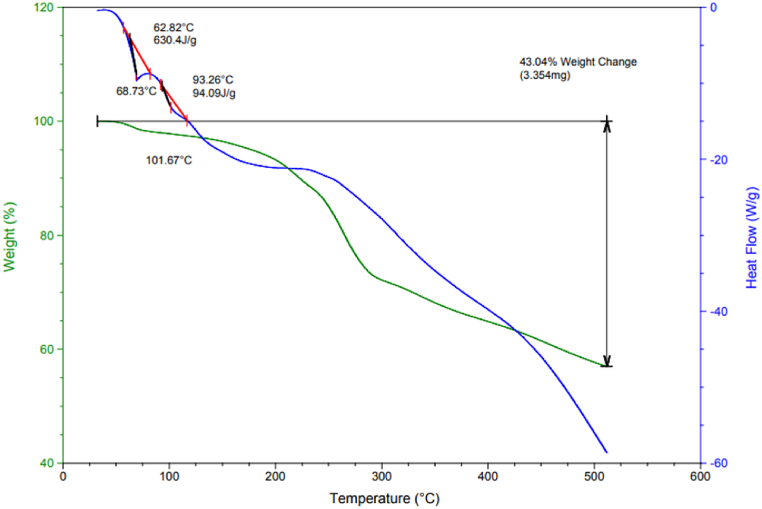
TGA and DSC thermal analyzes of LDH@PTRMS@BDSA@Cu(NO_3_)_2_ catalyst.

Thermal stability analysis, conducted via thermogravimetric analysis (TGA) and differential scanning calorimetry (DSC), is presented in Fig. 3. TGA reveals several stages of mass reduction with increasing temperature: initial mass loss below 100 °C attributed to water desorption mainly from the layers, subsequent mass loss around 290 °C associated with organic group decomposition and dissolution, and mass loss beyond 430 °C indicative of catalyst decomposition. Moreover, DSC corroborates the findings of TGA analysis.

**Fig. 4:**
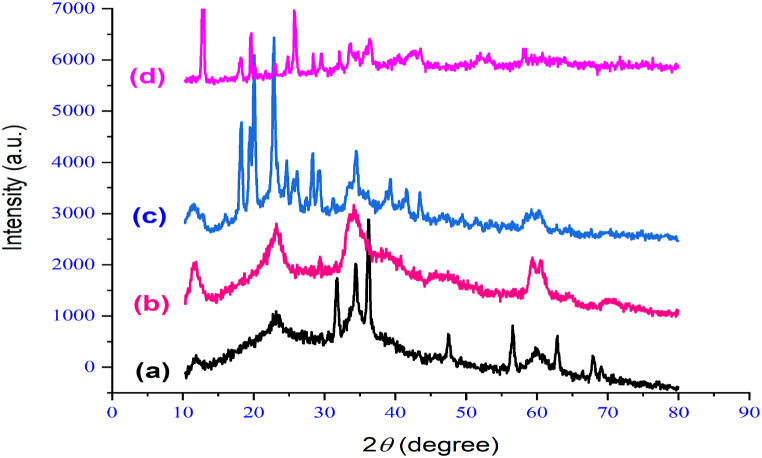
X-ray diffraction of the catalyst and its intermediates, (a) LDH, (b) LDH@PTRMS, (c) LDH@PTRMS@BDSA and (d) LDH@PTRMS@BDSA@Cu(NO_3_)_2_

XRD examination was employed to explore both the crystalline structure and dimensions of the catalyst particles. In Fig. 4, XRD patterns from various stages of nano-catalyst synthesis are depicted [44]. Part d, representing the final catalyst stage, exhibits peaks at 21/12^⸰^, 25/29^⸰^, 31/78^⸰^, 33/31^⸰^, 36/26^⸰^, 39^⸰^, 41^⸰^, 41/43^⸰^, 49^⸰^, 51/25^⸰^, 53/35^⸰^, and 57.98^⸰^, associated with copper nitrate. Other peaks correspond to the layered double hydroxides and 1,3-benzenedisulfonyl amide ligand, indicating high crystallinity and long-range order in these samples. The XRD difference between LDH and LDH@PTRMS can be attributed to the presence of 3-chloropropyltrimethoxysilane on the LDH surface, which alters the XRD pattern of LDH. The changes observed in part C are due to the adhesion of the BDSA ligand on the surface of LDH@PTRMS, while in part D, they result from the placement of copper nitrate on the surface of LDH@PTRMS@BDSA. These modifications result in a complete change in the XRD pattern, indicating the correct arrangement of the layers.

**Fig. 5:**
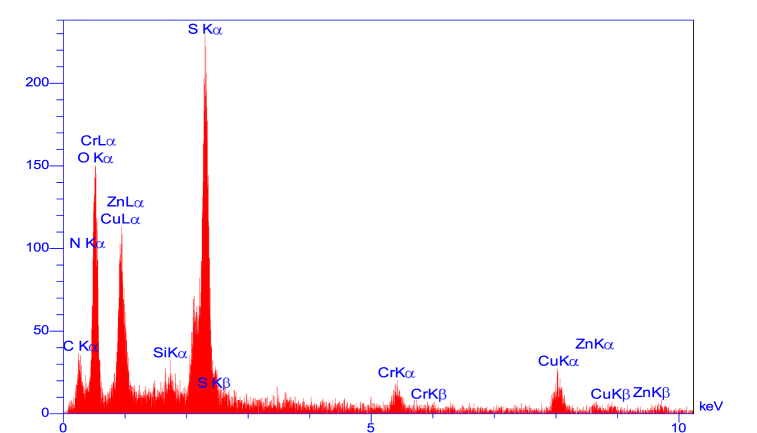
EDX analysis of LDH@PTRMS@BDSA@Cu(NO_3_)_2_ catalyst.

**Fig. 6:**
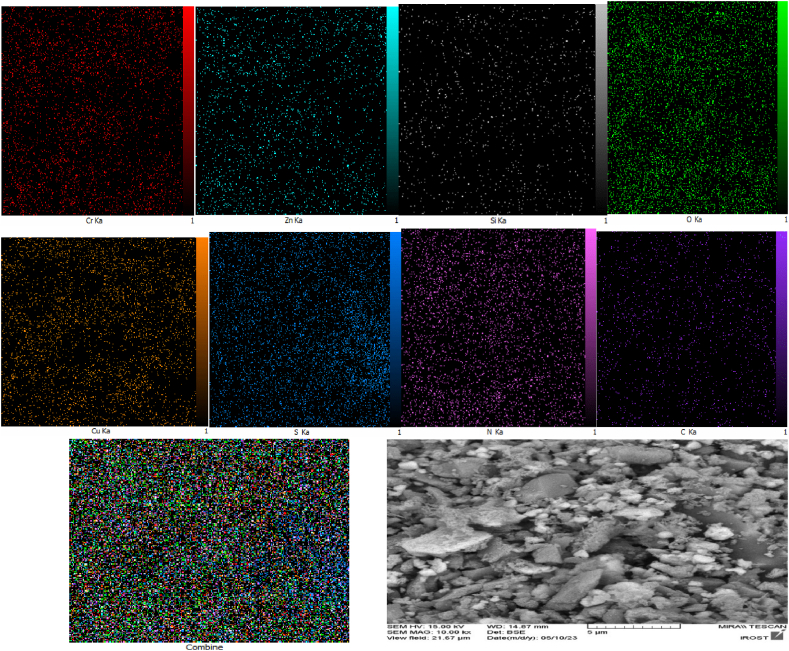
Elemental mapping analysis of LDH@PTRMS@BDSA@Cu(NO_3_)_2_ catalyst.

Furthermore, in a separate investigation, EDX analysis was employed to assess the chemical properties and elemental composition of the catalyst. The findings of this examination validated the presence of oxygen, sulfur, nitrogen, silicon, carbon, copper, zinc, and chromium elements within the catalyst structure (refer to Fig. 5). Additionally, as depicted in Fig. 6, elemental analysis (mapping) of the composition reaffirms the presence of the aforementioned elements in the LDH@PTRMS@BDSA@Cu(NO_3_)_2_ catalyst.

### 3.2 Catalytic application of LDH@PTRMS@BDSA@Cu(NO_3_)_2_ in the preparation of pyrido[2,3-*d*]pyrimidine derivatives

Following the confirmation of the new nano catalyst through the aforementioned methods, its catalytic efficacy was assessed in the one-pot three-component reaction of pyrido[2,3-*d*]pyrimidine derivatives. Initially, to determine optimal conditions, a model reaction utilizing malononitrile (1 mmol), 6-amino-1,3-dimethylpyrimidine-2,4(1*H*,3*H*)-diene (1 mmol), and 4-chlorobenzaldehyde (1 mmol) as substrates was conducted. A range of solvents were utilized, comprising H_2_O, ethanol, H_2_O-ethanol mixtures, methanol, acetonitrile, and conditions devoid of solvents at different temperatures and catalyst concentrations. Results revealed that polar solvents such as ethanol and H_2_O/ethanol exhibited favorable effects on product yield. Optimal conditions, yielding the highest efficiency with a short reaction time, were achieved using a mixture of water and ethanol (0.5:0.5 mL) at 70 °C in the presence of 0.05 g of LDH@PTRMS@BDSA@Cu(NO_3_)_2_ nanoparticles. The summarized findings are presented in Table 1. LDH@PTRMS@BDSA@Cu(NO_3_)_2_ emerged as a suitable catalyst for synthesizing pyrido[2,3-*d*]pyrimidine derivatives, demonstrating both short reaction times and high efficiency.

**Table 1:**
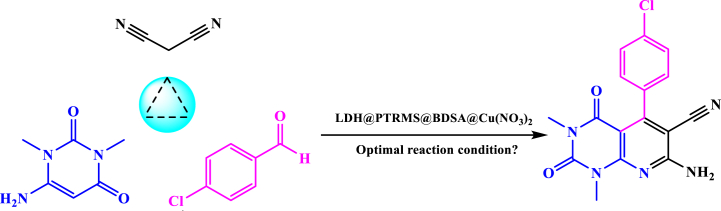
Optimization of reaction conditions for the synthesis of 7-amino-5-(4-chlorophenyl)-1,3-dimethyl-2,4-dioxo-1,2,3,4-tetrahydropyrido[2,3-*d*]pyrimidine-6-carbonitrile.

Entry	Solvent	Load of catalyst (g)	Temperature (°C)	Time (min)	Yield (%)
1	–	–	100	60	Trace
2	–	0.05	70	20	84
3	EtOH	0.05	reflux	30	30
4	H2O	0.05	reflux	50	Trace
5	H2O/EtOH (1:1)	0.05	reflux	15	92
6	MeOH	0.05	reflux	50	28
7	EtOAc	0.055	reflux	55	38
8	CH3CN	0.05	reflux	45	Trace
9	H2O/EtOH (1:1)	0.05	90	12	93
10	H2O/EtOH (1:1)	0.05	80	12	92
11	H2O/EtOH (1:1)	0.015	70	50	52
12	H2O/EtOH (1:1)	0.025	70	34	78
13	H2O/EtOH (1:1)	0.035	70	30	84
14	H2O/EtOH (1:1)	0.05	70	16	92
15	H2O/EtOH (1:1)	0.055	70	15	92

As indicated in Table 1, the absence of LDH@PTRMS@BDSA@Cu(NO_3_)_2_ nano catalysts led to prolonged reaction times, higher temperatures, and decreased efficiency. Various temperatures ranging from room temperature to 90 °C were explored, with 70 °C identified as the optimal reaction condition. Subsequently, the optimal reaction conditions for synthesizing pyrido[2,3-*d*]pyrimidine derivatives using 6-amino-1,3-dimethylpyrimidine-2,4(1*H*,3*H*)-dione, malononitrile, and arylaldehydes featuring different electron-donating or electron-withdrawing groups were determined (Table 2).

**Table 2:**
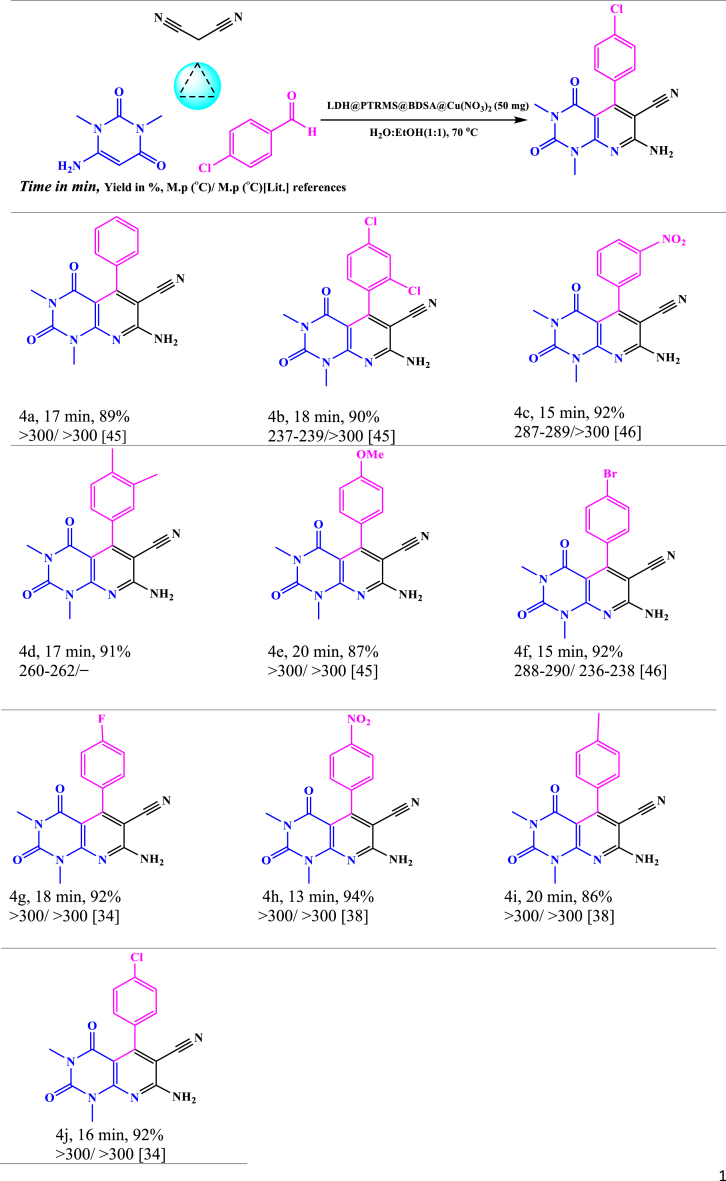
Synthesis of pyrido[2,3-*d*]pyrimidine derivatives in the vicinity of LDH@PTRMS@BDSA@Cu(NO_3_)_2_ catalyst.

As summarized in Table 2, all pyrido[2,3-*d*]pyrimidine derivatives were readily synthesized with exceptional yields, affirming the remarkably high catalytic activity of LDH@PTRMS@BDSA@Cu(NO_3_)_2_ nano-catalysts for pyrido[2,3-*d*]pyrimidine synthesis.

**Fig. 7:**
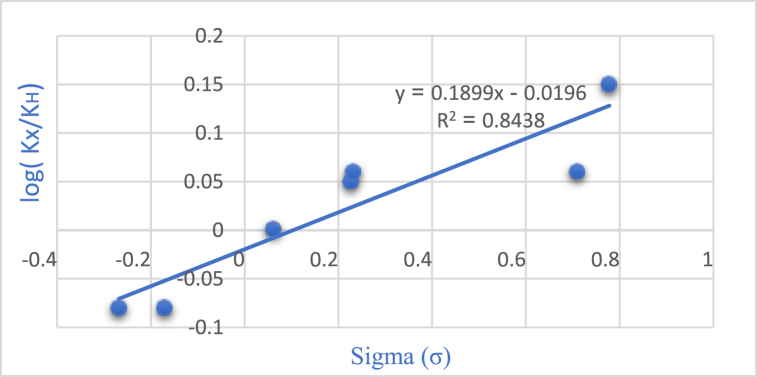
Investigating the Substitution Groups in pyrido[2,3-*d*]pyrimidine Synthesis through the Hammett Plot.

The Hammett equation, a linear free energy equation, elucidates the relationship between reaction energetics and substituent effects. Alterations in the free energy of the reaction correspondingly affect the activation energy of the transition state. By plotting the ratio of rate constants Log(k_X_/k_H_) against the substituent constant (σ) in for various substituent groups situated meta and para to the reaction center, as illustrated in Fig. 7 of the Hammett diagram, we discern distinct patterns. Notably, the positive slope observed in the Hammett reaction diagram indicates that electron-withdrawing groups enhance the reaction rate.

**Scheme 2:**
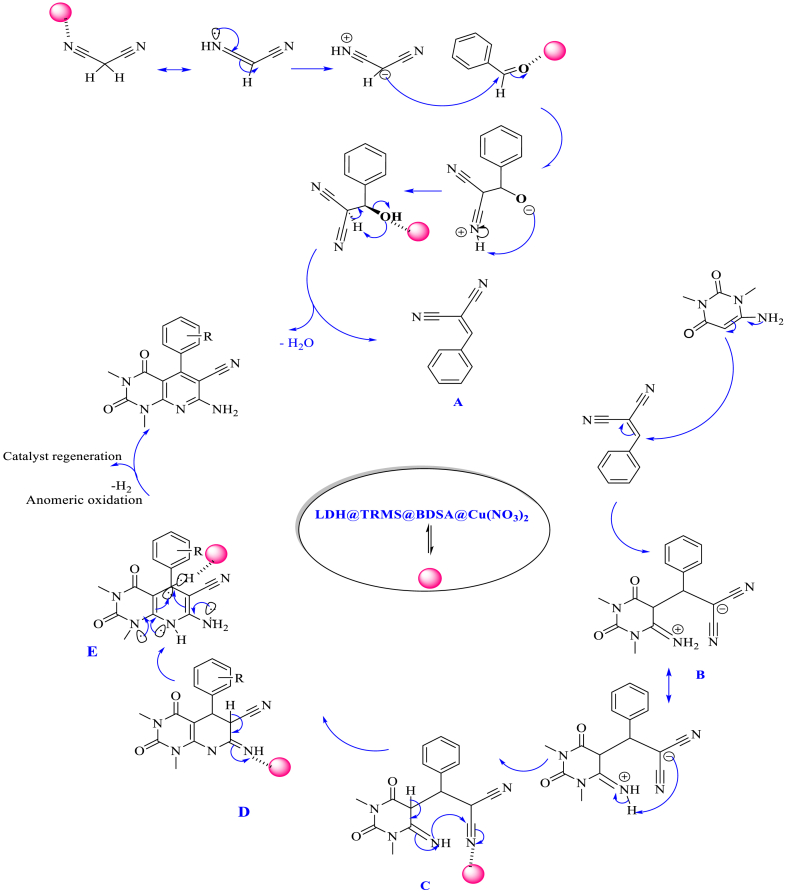
The proposed mechanism for the synthesis of pyrido[2,3-*d*]pyrimidine derivatives in the vicinity of LDH@PTRMS@BDSA@Cu(NO_3_)_2_ catalyst.

The mechanism proposed for synthesizing pyrido[2,3-*d*]pyrimidine derivatives in the presence of LDH@PTRMS@BDSA@Cu(NO_3_)_2_ catalyst is illustrated in Scheme 2. Initiated by the activation of carbonyl benzaldehyde by the nano catalyst, it becomes susceptible to nucleophilic attack by activated malononitrile, resulting in intermediate A. This intermediate then reacts with 6-amino-1,3-dimethylpyrimidine-2,4(1*H*,3*H*)-dione to form intermediate B. By tautomerization of intermediate B, intermediate C is formed. Facilitated by the catalyst, intermediate C undergoes intramolecular attack to yield intermediate D. Intermediate E is formed by tautomerization of intermediate D. Subsequently, through anomeric oxidation, a hydrogen molecule (H_2_) is released, and the product is synthesized via the tautomerization of intermediate E in close proximity to the catalyst. The catalyst is then the LDH@PTRMS@BDSA@Cu(NO_3_)_2_ nanoparticles were easily separated through simple extraction, recycled and employed in subsequent cycles.

**Fig. 8:**
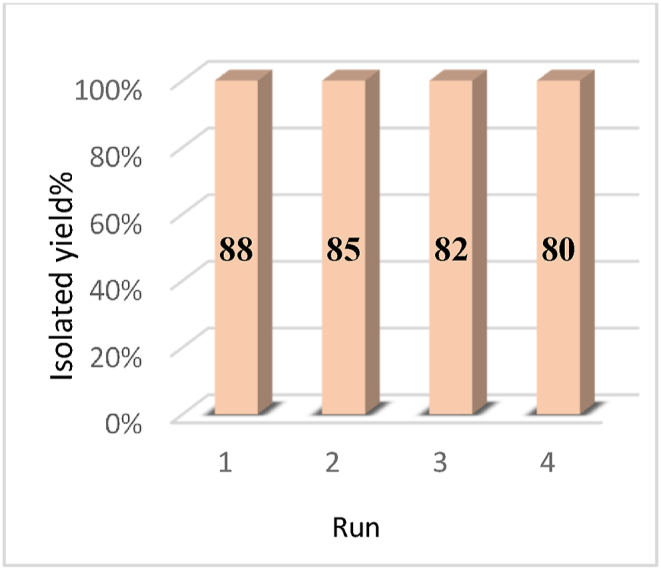
Ability to recycle and reuse LDH@PTRMS@BDSA@Cu(NO_3_)_2_ catalyst.

Investigations were conducted into the recycling and reuse of catalysts in line with green chemistry principles. To this end, LDH@PTRMS@BDSA@Cu(NO_3_)_2_ nanoparticles were recovered under optimal reaction conditions involving malononitrile (1 mmol), 6-amino-1,3-dimethylpyrimidine-2,4(1*H*,3*H*)-dione (1 mmol), and 4-chlorobenzaldehyde (1 mmol) in a water and ethanol solvent mixture (0.5:0.5 mL) at 70 °C, with 0.05 g of catalyst. Following each cycle, the nano catalyst was separated by adding 3 mL of hot ethanol or chloroform to the reaction mixture, stirring magnetically for 2 min, and subsequently centrifuging. It underwent three washes with ethanol before being dried in an oven at 60 °C for reuse in the subsequent cycle. As depicted in Fig. 8, the catalyst exhibited recyclability for four consecutive cycles, maintaining acceptable activity throughout each cycle without significant reduction in its catalytic efficiency.

According to the data presented in Table 3, the LDH@PTRMS@BDSA@Cu(NO_3_)_2_ catalytic method was juxtaposed with previously reported protocols for synthesizing pyrido[2,3-*d*]pyrimidine s. The results underscored the efficacy of this novel catalyst, which demonstrated superior performance in terms of efficiency, reaction time, and operating temperature. These findings highlight the remarkable efficiency of the new catalyst in comparison to alternative catalysts.

**Table 3:** Comparing the efficiency of LDH@PTRMS@BDSA@Cu(NO_3_)_2_ catalyst in the synthesis of pyrido[2,3-*d*]pyrimidine derivatives with some other catalysts.

Entry	Reaction conditions	Time (min)	Yield (%)	Lit.
1	nanocrystalline MgO, H_2_O, 80 °C	30	90	[34]
2	MDW, 90 °C	90	88	[45]
3	Sodium p-toluene sulfonate (NaPTS), 28 °C	25	88	[46]
4	SBA-15/PrN(CH_2_PO_3_H_2_)_2_, solvent-free, 100 °C	5	85	[38]
5	Agar-entrapped [DABCO](SO_3_H)_2_Cl_2_, EtOH. reflux	60	94	[47]
6	Triethanolamine/H2O, 80 °C	120	92	[48]
7	Al-HMS-20/EtOH-r.t.	720	92	[36]
8	[H_2_-DABCO][ClO_4_]_2_/EtOH, 70 °C	50	95	[49]
9	LDH@PTRMS@BDSA@Cu(NO_3_)_2_, EtOH/H_2_O (1:1), 70 °C	16	92	This work

## 4 Conclusion

In summary, an innovative method to develop a new LDH@PTRMS@BDSA@Cu(NO_3_)_2_ nano catalyst was introduced. Comprising copper acetate species anchored onto a nanocomposite derived from a modified layered double hydroxide with 1,3-benzenedisulfonyl amide, this catalyst underwent comprehensive validation via various instrumental techniques (FTIR, SEM, TGA, DSC, XRD, EDX, and MAPPING). Subsequently, it proved highly effective and recyclable when employed as a new nano catalyst in a one-pot three-component reaction for synthesizing pyrido[2,3-*d*]pyrimidine compounds under mild conditions. It is worth mentioning that this method offers a green chemistry path by removing toxic organic solvents. Key advantages of this method include its utilization of readily available raw materials, streamlined one-pot reaction process, environmental friendliness, ease of product purification, short reaction duration, and high efficiency. Moreover, the catalyst can be readily extracted from the reaction mixture and reused up to four times without significant efficiency loss.

## CRediT authorship contribution statement

**Sarieh Momeni:** Writing – review & editing, Writing – original draft, Funding acquisition, Formal analysis, Data curation. **Ramin Ghorbani-Vaghei:** Writing – review & editing, Supervision, Project administration, Methodology.

## Data availability

Data will be made available on request.

## Declaration of competing interest

The authors declare that they have no known competing financial interests or personal relationships that could have appeared to influence the work reported in this paper.
