# Supplementary material for: High-performance modified LDH for green one-pot synthesis of pyrido[2,3-d]pyrimidines
Source: Heliyon. 2024 Dec 12;11(1):e41149. doi: 10.1016/j.heliyon.2024.e41149 (PMC11714396; doi:10.1016/j.heliyon.2024.e41149)
Supplement: Multimedia component 1 [file mmc1.docx]

*Supplementary Information (SI)*

**High-performance modified LDH for green one-pot synthesis of** **pyrido[2,3-*d*]pyrimidines**

Sarieh Momeni^1^, Ramin Ghorbani-Vaghei^1,2^ *

Department of Organic Chemistry, Faculty of Chemistry and Petroleum Sciences, Bu-Ali Sina University, 6517838683, Hamadan, *Iran*

^2^Department of Organic Chemistry, Faculty of Chemistry, Guilan University, Rasht, Iran

**Corresponding author; E-mail:* [*rgvaghei@yahoo.com*](mailto:rgvaghei@yahoo.com) *&* [*ghorbani@basu.ac.ir*](mailto:ghorbani@basu.ac.ir)

**Spectral Data:**

**7-amino-5-(2,4-dichlorophenyl)-1,3-dimethyl-2,4-dioxo-1,2,3,4-tetrahydropyrido[2,3-d]pyrimidine-6-carbonitrile (4b)**

Melting point=190-192 °C,

FT-IR (KBr, ν cm^-1^): 3467, 3332, 3225, 3103, 2229, 1712, 1667, 1626, 1579, 1437, 1373, 1109, 1051, 867, 823, 581; ^1^H NMR (250 MHz, CDCl_3_) δ ^1^H NMR (250 MHz, Chloroform-d) δ 7.54 (s, 1H), 7.39 (d, J = 8.2 Hz, 1H), 7.12 (d, J = 8.3 Hz, 1H), 5.79 (s, 2H), 3.65 (s, 3H), 3.30 (s, 3H); ^13^C NMR (63 MHz, DMSO-*d_6_*) δ 13C NMR (63 MHz, CDCl_3_) δ 160.1, 158.8, 155.8, 153.9, 151.0, 135.7, 134.0, 132.26, 129.7, 128.9, 127.5, 114.4, 100.6, 89.6, 35.5, 34.8, 30.2, 28.3.


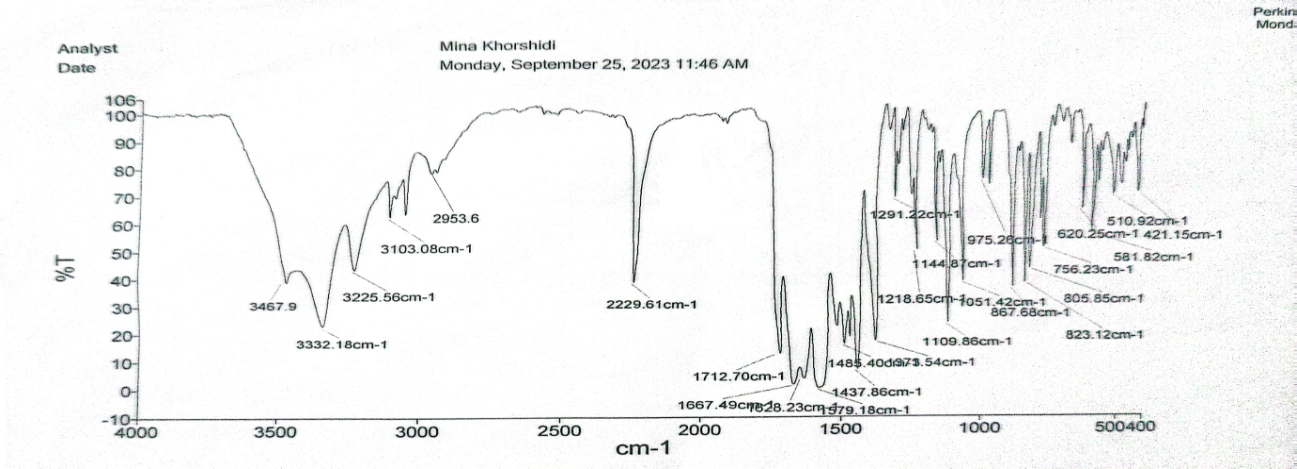


*Figure S1. Spectrum FTIR of 7-amino-5-(2,4-dichlorophenyl)-1,3-dimethyl-2,4-dioxo-1,2,3,4-tetrahydropyrido[2,3-d]pyrimidine-6-carbonitrile*


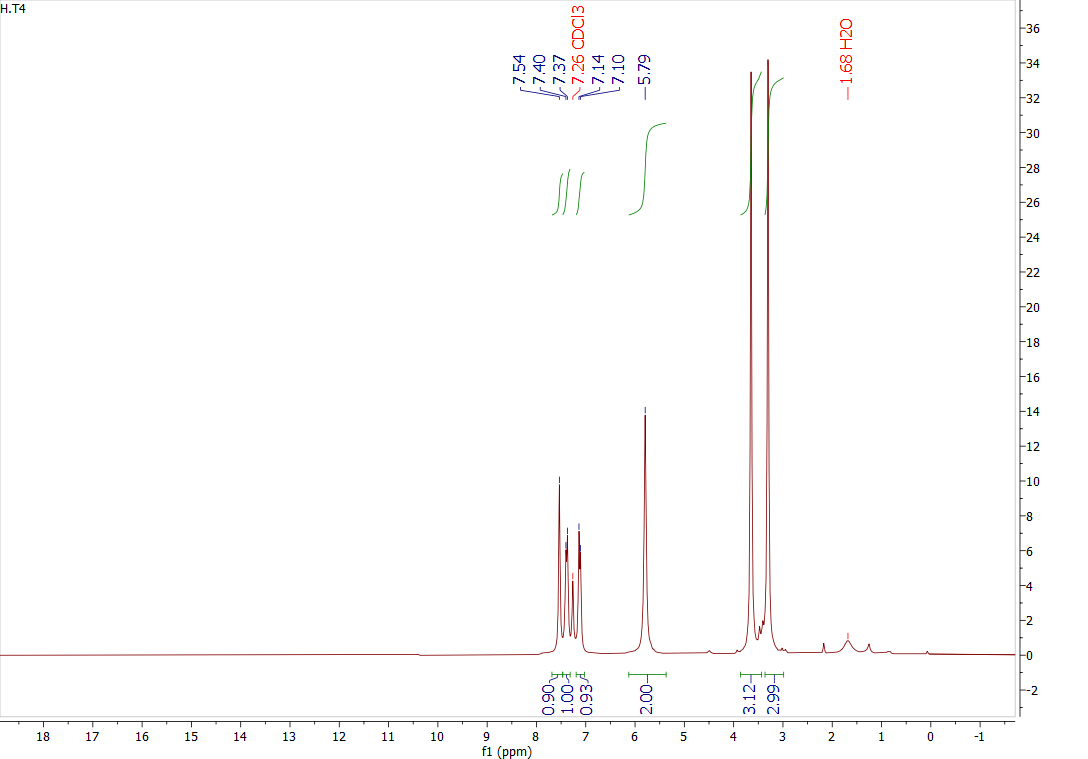


*Figure S2. Spectrum ^1^H NMR (250 MHz) of 7-amino-5-(2,4-dichlorophenyl)-1,3-dimethyl-2,4-dioxo-1,2,3,4-tetrahydropyrido[2,3-d]pyrimidine-6-carbonitrile in CDCl_3_ solvent*


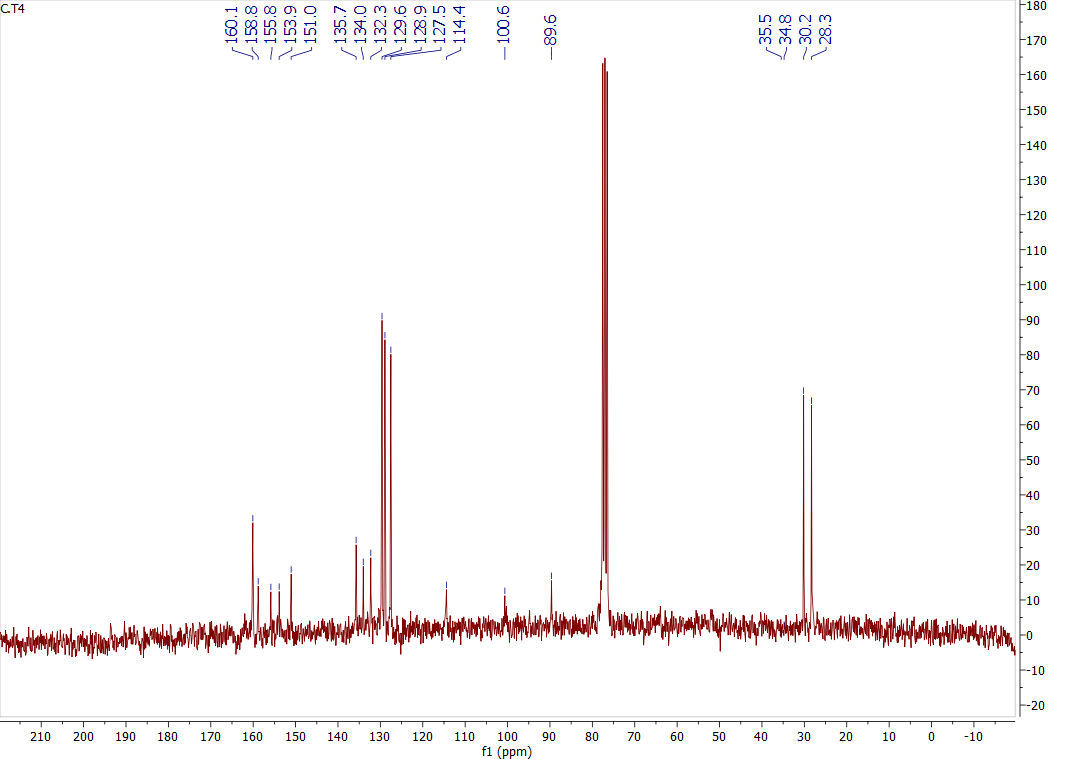


*Figure S3. Spectrum ^13^C NMR (63 MHz) of 7-amino-5-(2,4-dichlorophenyl)-1,3-dimethyl-2,4-dioxo-1,2,3,4-tetrahydropyrido[2,3-d]pyrimidine-6-carbonitrile in CDCl_3_ solvent*

**7-amino-1,3-dimethyl-5-(3-nitrophenyl)-2,4-dioxo-1,2,3,4-tetrahydropyrido[2,3-d]pyrimidine-6-carbonitrile (4c)**


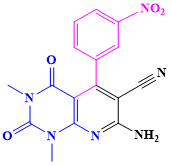


Melting point=287-289 °C,

FT-IR (KBr, ν cm^-1^): 3460, 3336, 3232, 3066, 2966, 2226, 1711, 1667, 1626, 1565, 1532, 1441, 1361, 1231, 1095, 805, 699, 502; ^1^H NMR (250 MHz, CDCl_3_) δ ^1^H NMR (250 MHz, Chloroform-d) δ 8.53 – 8.23 (m, 1H), 8.15 (s, 1H), 7.70 (t, J = 7.8 Hz, 1H), 7.60 (d, J = 8.0 Hz, 1H), 5.81 (s, 2H), 3.66 (s, 3H), 3.30 (d, J = 8.0 Hz, 3H); ^13^C NMR (63 MHz, CDCl_3_) δ 13C NMR (63 MHz, CDCl3) δ 159.9, 157.1, 155.4, 151.0, 148.0, 137.7, 135.3, 133.1, 129.5, 124.0, 122.7, 114.5, 30.3, 28.3.


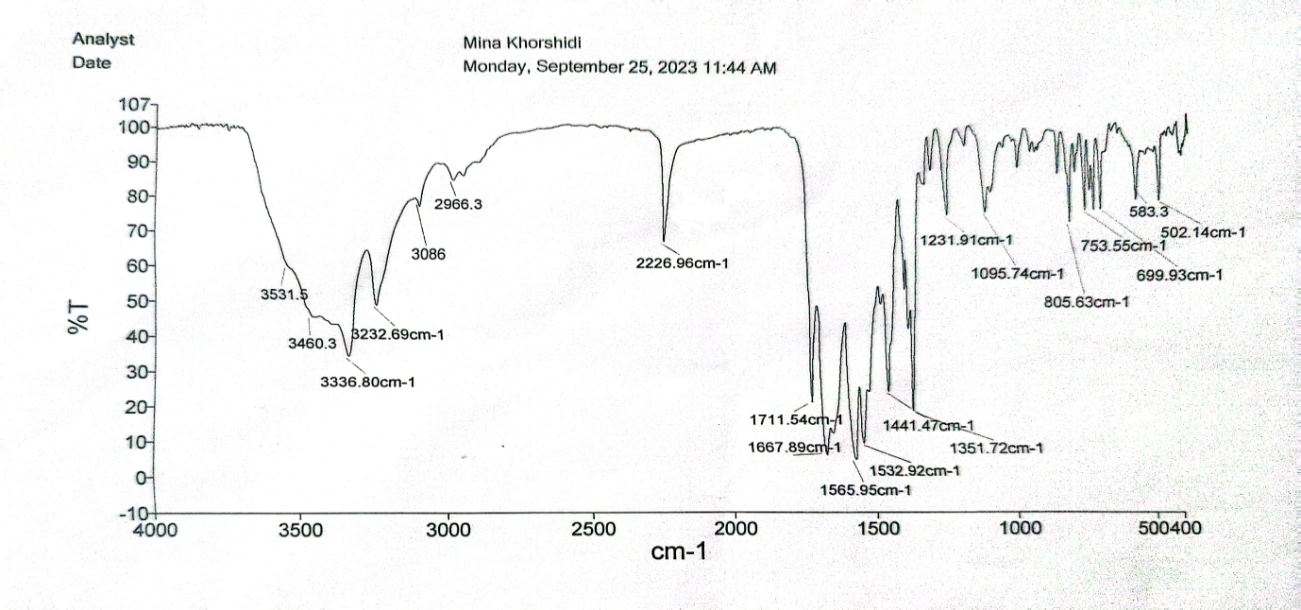


*Figure S4. Spectrum FTIR of 7-amino-1,3-dimethyl-5-(3-nitrophenyl)-2,4-dioxo-1,2,3,4-tetrahydropyrido[2,3-d]pyrimidine-6-carbonitrile*


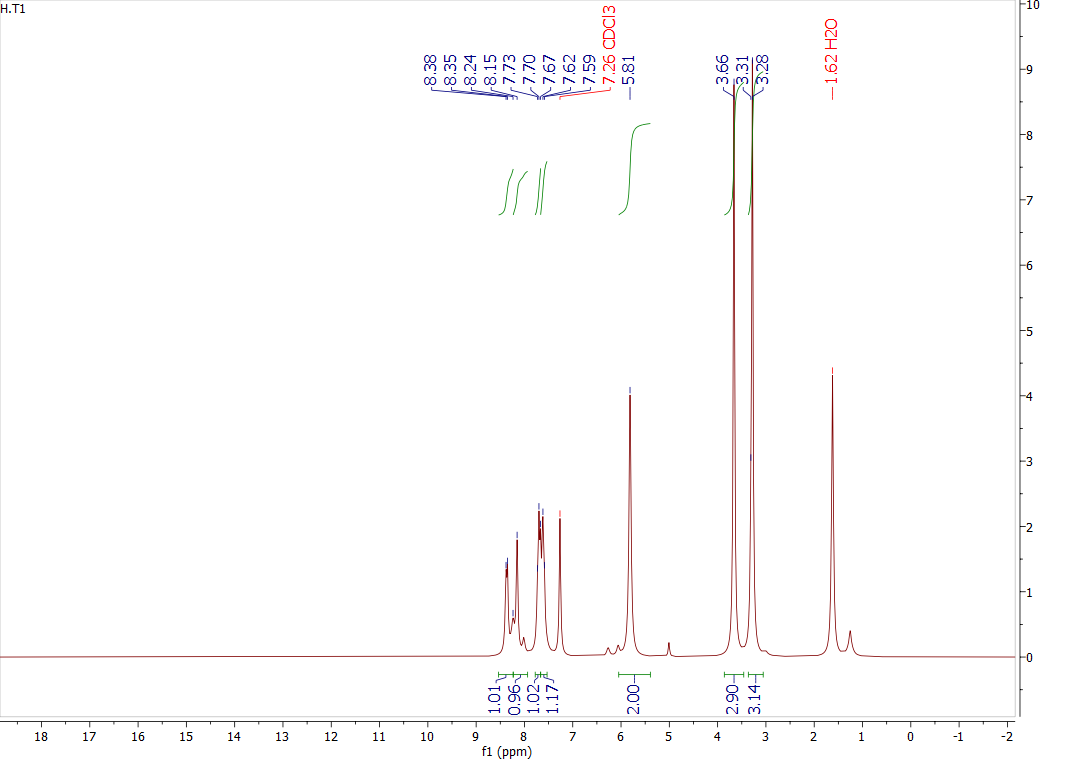


*Figure S5. Spectrum ^1^H NMR (250 MHz) of 7-amino-1,3-dimethyl-5-(3-nitrophenyl)-2,4-dioxo-1,2,3,4-tetrahydropyrido[2,3-d]pyrimidine-6-carbonitrile in CDCl_3_ solvent*


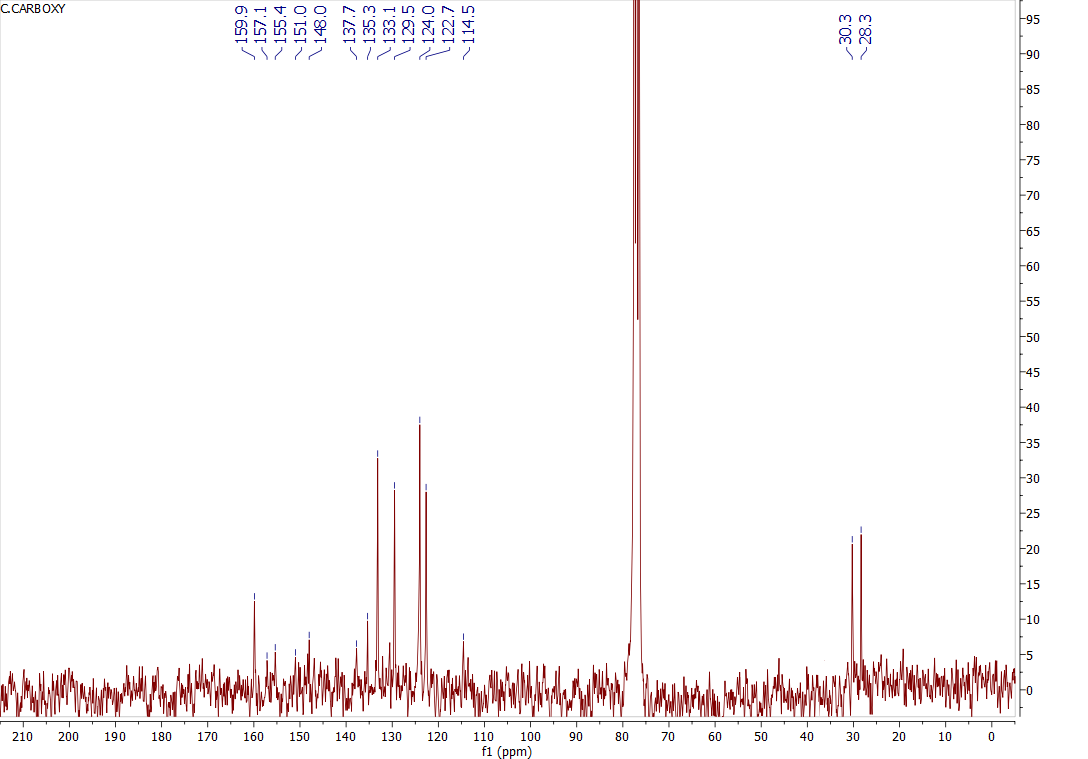


*Figure S6. Spectrum ^13^C NMR (63 MHz) of 7-amino-1,3-dimethyl-5-(3-nitrophenyl)-2,4-dioxo-1,2,3,4-tetrahydropyrido[2,3-d]pyrimidine-6-carbonitrile in CDCl_3_ solvent*

**7-amino-5-(3,4-dimethylphenyl)-1,3-dimethyl-2,4-dioxo-1,2,3,4-tetrahydropyrido[2,3-d]pyrimidine-6-carbonitrile (4d)**

**
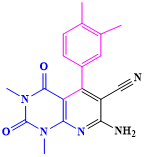
**

Melting point=260-262 °C,

FT-IR (KBr, ν cm^-1^): 3472, 3396, 3348, 3226, 2955, 2224, 1712, 1662, 1636, 1570, 1485, 1370, 1101, 1051, 804, 754, 565; ^1^H NMR (250 MHz, CDCl_3_) δ ^1^H NMR (250 MHz, Chloroform-d) δ 7.48 (d, J = 107.9 Hz, 1H), 6.99 (d, J = 12.0 Hz, 2H), 5.70 (s, 2H), 3.64 (s, 3H), 3.30 (s, 3H), 2.33 (s, 6H); ^13^C NMR (63 MHz, DMSO-*d_6_*) δ 13C NMR (63 MHz, CDCl_3_) δ 160.7, 159.9, 159.0, 153.9, 151.3, 137.7, 136.5, 133.7, 129.6, 128.0, 124.5, 115.4, 100.5, 90.3, 30.2, 28.3, 20.0, 19.8.


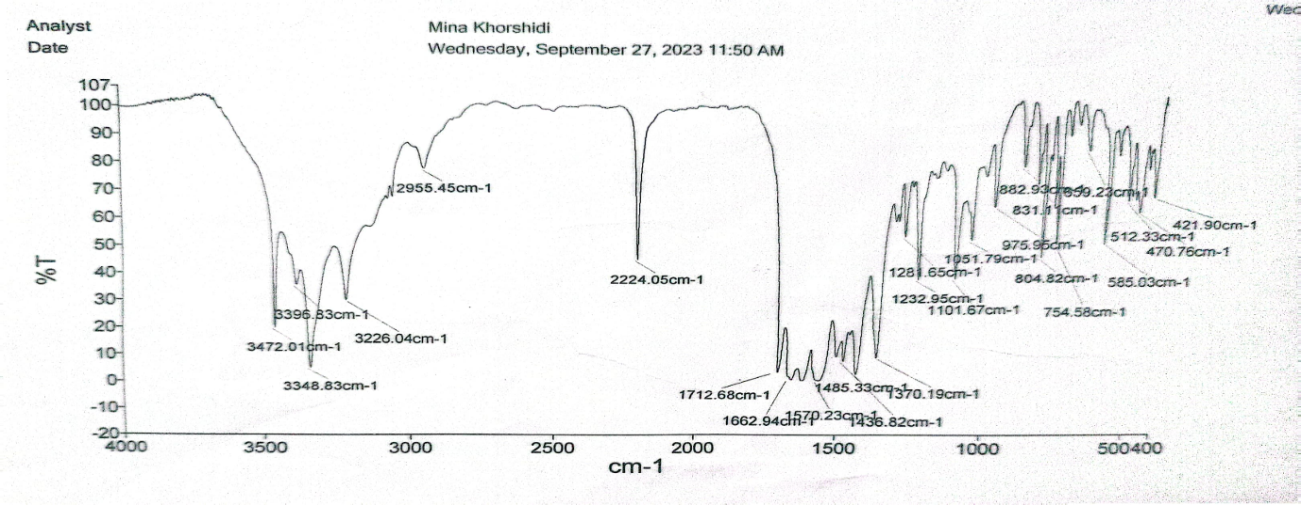


*Figure S7. Spectrum FTIR of* *7-amino-5-(3,4-dimethylphenyl)-1,3-dimethyl-2,4-dioxo-1,2,3,4-tetrahydropyrido[2,3-d]pyrimidine-6-carbonitrile*


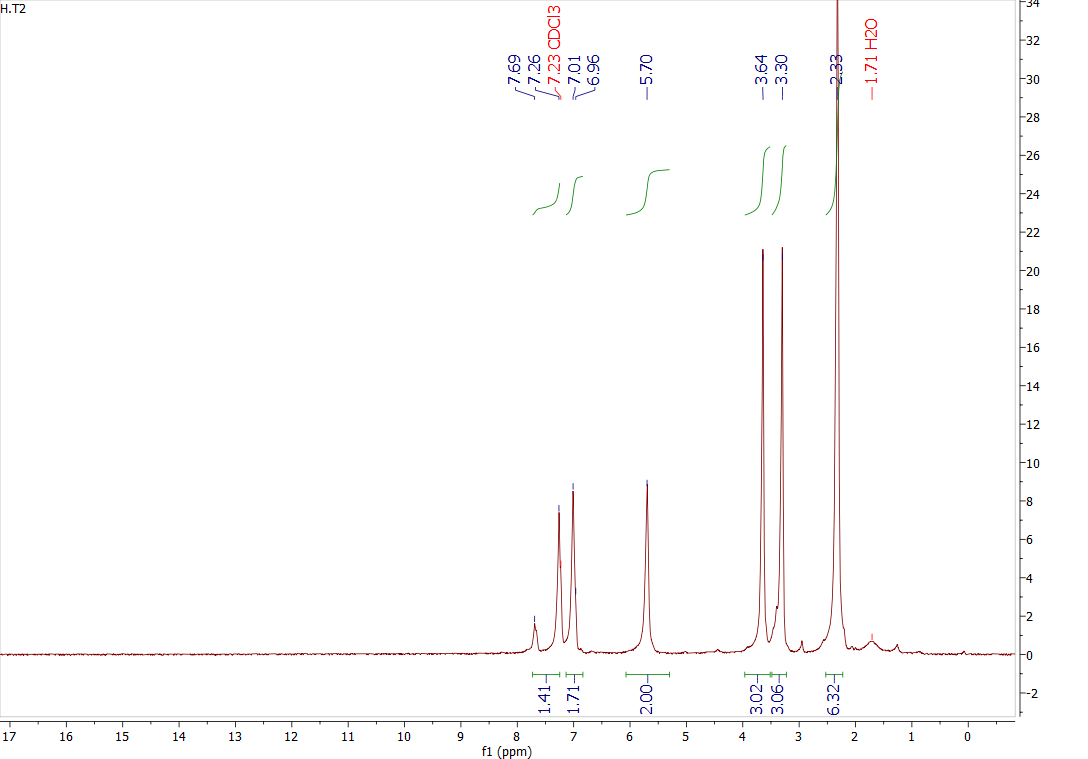


*Figure S8. Spectrum ^1^H NMR (250 MHz) of 7-amino-5-(3,4-dimethylphenyl)-1,3-dimethyl-2,4-dioxo-1,2,3,4-tetrahydropyrido[2,3-d]pyrimidine-6-carbonitrile in CDCl_3_ solvent*


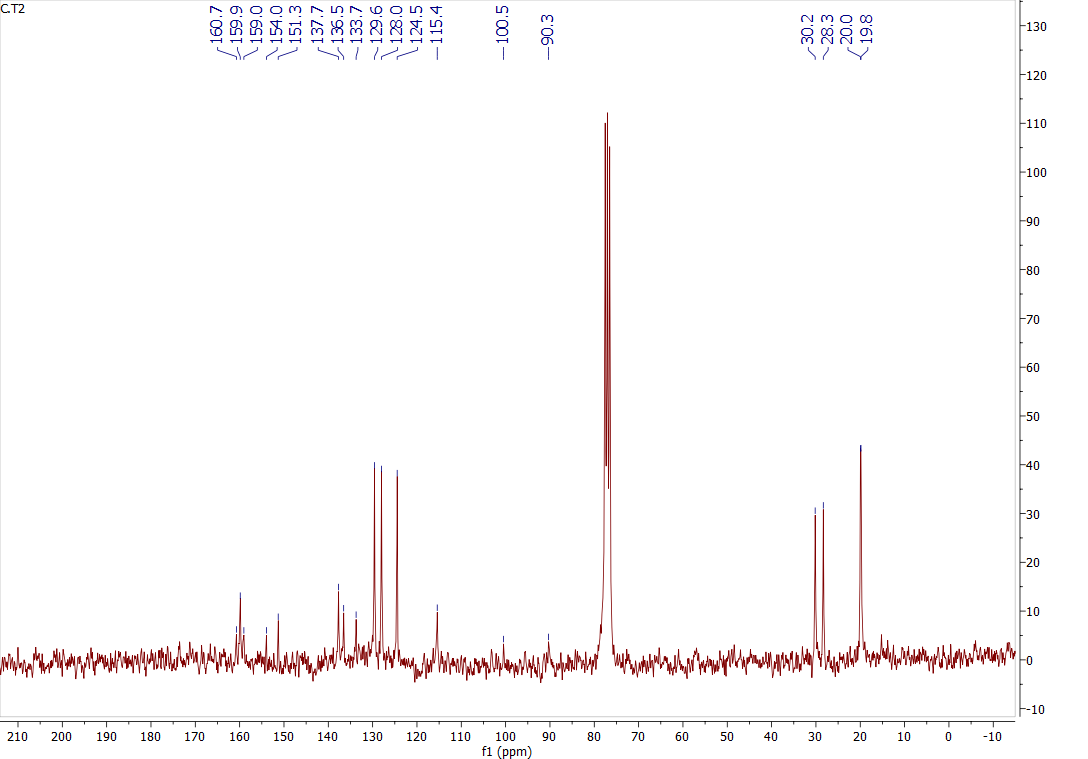


*Figure S9. Spectrum ^13^C NMR (63 MHz) of 7-amino-5-(3,4-dimethylphenyl)-1,3-dimethyl-2,4-dioxo-1,2,3,4-tetrahydropyrido[2,3-d]pyrimidine-6-carbonitrile in CDCl_3_ solvent*

**7-amino-5-(4-methoxyphenyl)-1,3-dimethyl-2,4-dioxo-1,2,3,4-tetrahydropyrido[2,3-d]pyrimidine-6-carbonitrile (4e)**


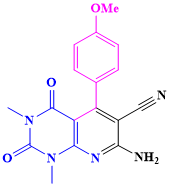


Melting point= >300 °C,

FT-IR (KBr, ν cm^-1^): 3462, 3315, 3221, 3056, 2215, 1698, 1667, 1623, 1585, 1551, 1468, 1441, 1364, 1015, 810, 791, 504; ^1^H NMR (250 MHz, CDCl_3_) δ ^1^H NMR (250 MHz, Chloroform-d) δ 7.25 (d, J = 8.5 Hz, 2H), 7.07 – 6.89 (m, 2H), 5.70 (s, 2H), 3.86 (s, 3H), 3.64 (s, 3H), 3.46 (s, 3H); ^13^C NMR (63 MHz, DMSO-*d_6_*) δ 13C NMR (63 MHz, CDCl_3_) δ 160.2, 159.9, 157. 6, 154.0, 151.0, 129.9, 128.7, 127.6, 113.70, 113.4, 101.2, 90.4, 55.2, 30.2, 28.3.


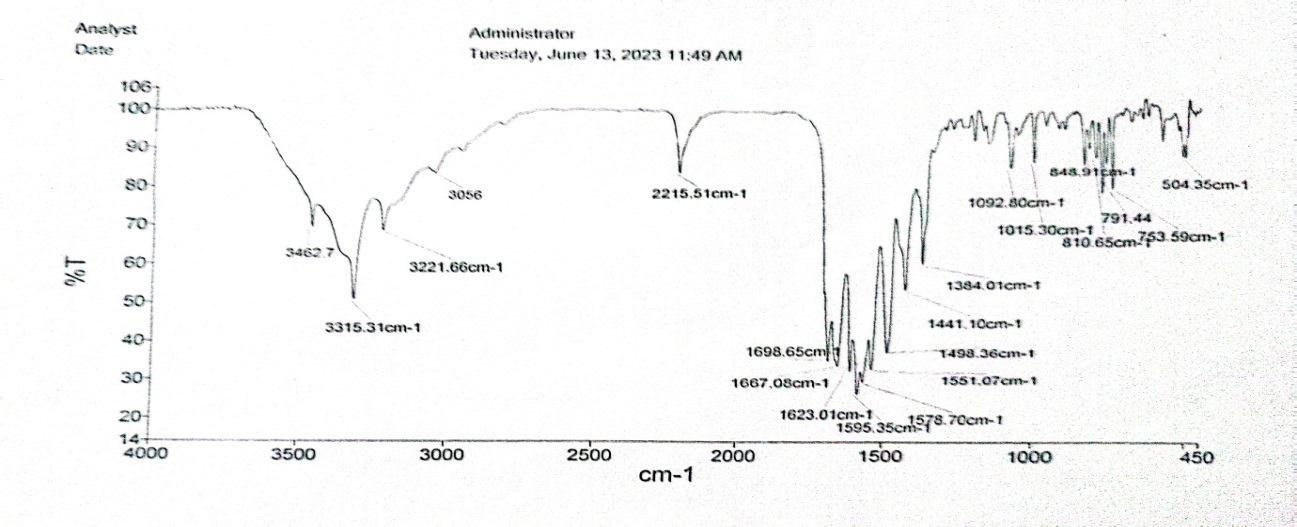


*Figure S10. Spectrum FTIR of 7-amino-5-(4-methoxyphenyl)-1,3-dimethyl-2,4-dioxo-1,2,3,4-tetrahydropyrido[2,3-d]pyrimidine-6-carbonitrile*


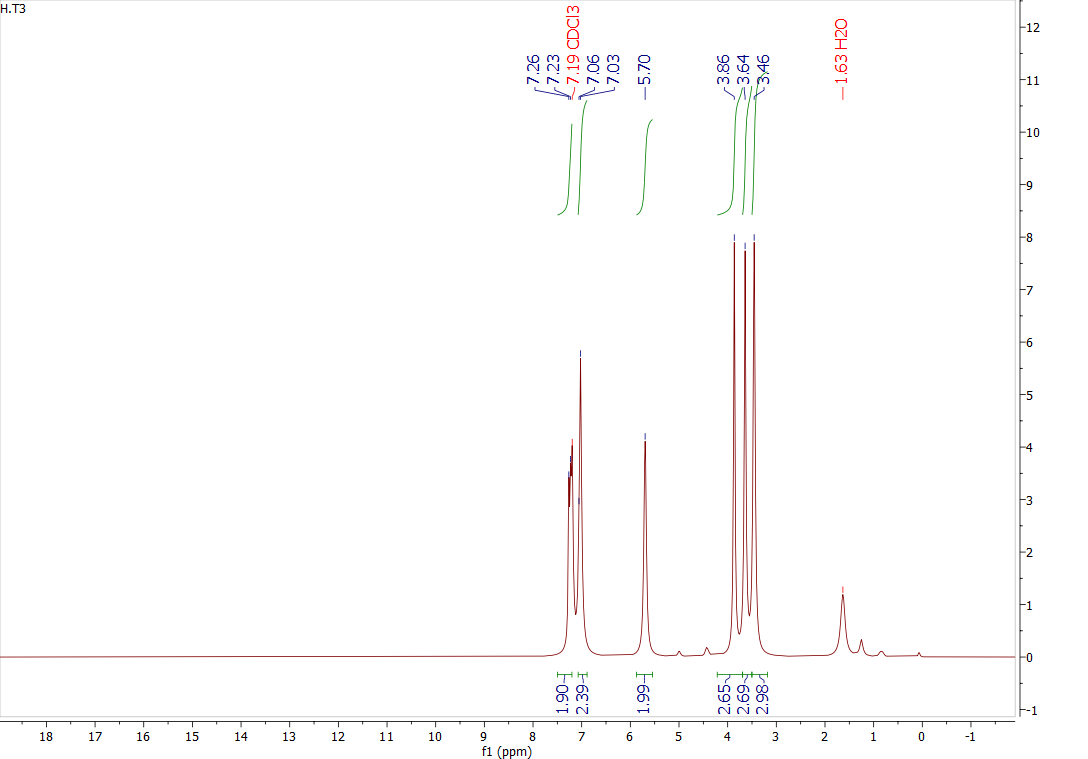


*Figure S11. Spectrum ^1^H NMR (250 MHz) of 7-amino-5-(4-methoxyphenyl)-1,3-dimethyl-2,4-dioxo-1,2,3,4-tetrahydropyrido[2,3-d]pyrimidine-6-carbonitrile in CDCl_3_ solvent*


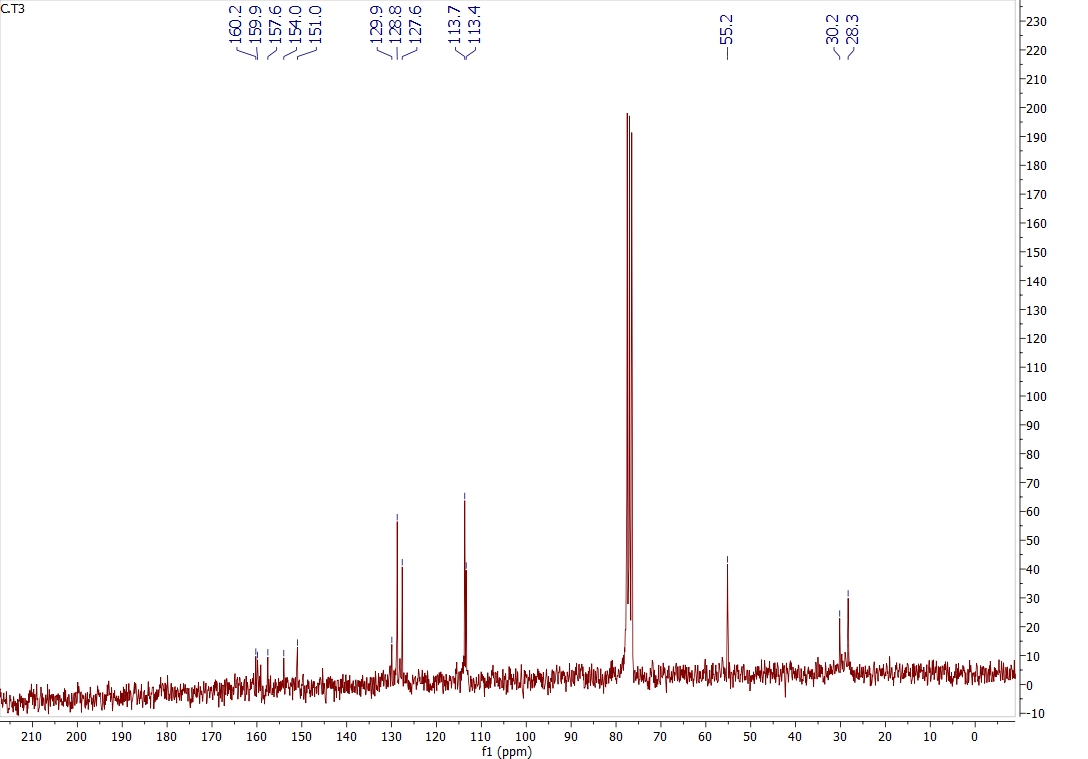


*Figure S12. Spectrum ^13^C NMR (63 MHz) of 7-amino-5-(4-methoxyphenyl)-1,3-dimethyl-2,4-dioxo-1,2,3,4-tetrahydropyrido[2,3-d]pyrimidine-6-carbonitrile in CDCl_3_ solvent*

**7-amino-5-(4-bromophenyl)-1,3-dimethyl-2,4-dioxo-1,2,3,4-tetrahydropyrido[2,3-d]pyrimidine-6-carbonitrile (4f)**


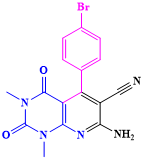


Melting point=288-290 °C,

FT-IR (KBr, ν cm^-1^): 3460, 3313, 3221, 3013, 2215, 1715, 1663, 1624, 1571, 1510, 1438, 1370, 1011, 830, 805, 580; ^1^H NMR (250 MHz, CDCl_3_) δ ^1^H NMR (250 MHz, Chloroform-d) δ 7.62 (d, J = 7.8 Hz, 2H), 7.14 (d, J = 8.0 Hz, 2H), 5.77 (s, 2H), 3.65 (s, 3H), 3.30 (s, 3H); ^13^C NMR (63 MHz, DMSO-*d_6_*) δ 13C NMR (63 MHz, CDCl_3_) δ 159.8, 159.0, 156.9, 154.0, 135.1, 132.1, 131.6, 131.0, 128.7, 123.6, 114.9, 106.7, 30.2, 28.3.


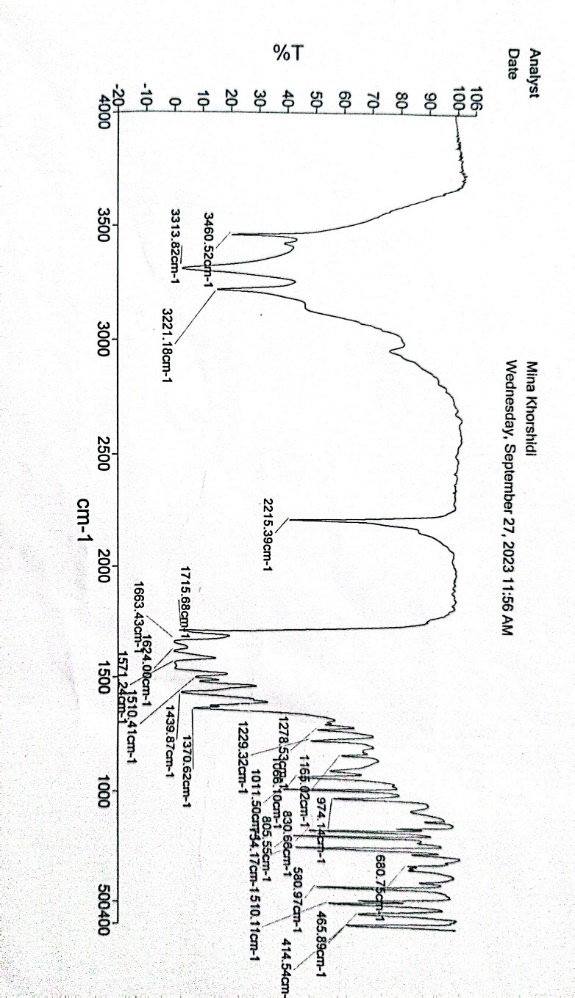


*Figure S16. Spectrum FTIR of 7-amino-5-(4-bromophenyl)-1,3-dimethyl-2,4-dioxo-1,2,3,4-tetrahydropyrido[2,3-d]pyrimidine-6-carbonitrile*


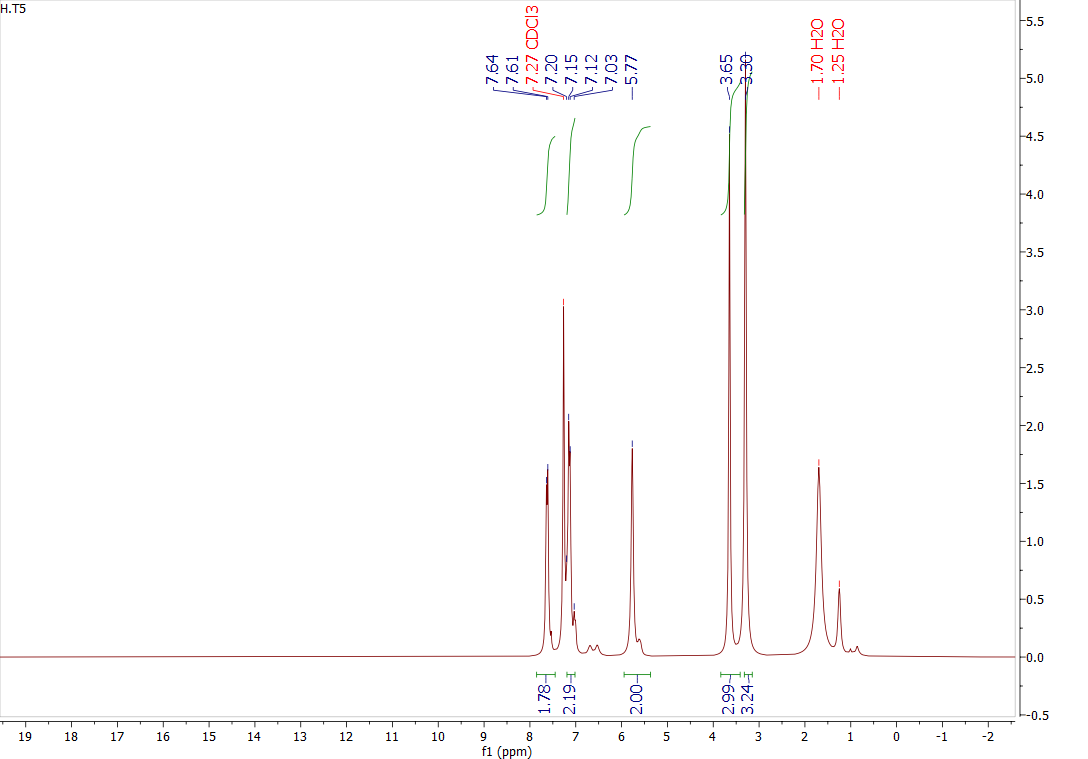


*Figure S17. Spectrum ^1^H NMR (250 MHz) of 7-amino-5-(4-bromophenyl)-1,3-dimethyl-2,4-dioxo-1,2,3,4-tetrahydropyrido[2,3-d]pyrimidine-6-carbonitrile in CDCl_3_ solvent*


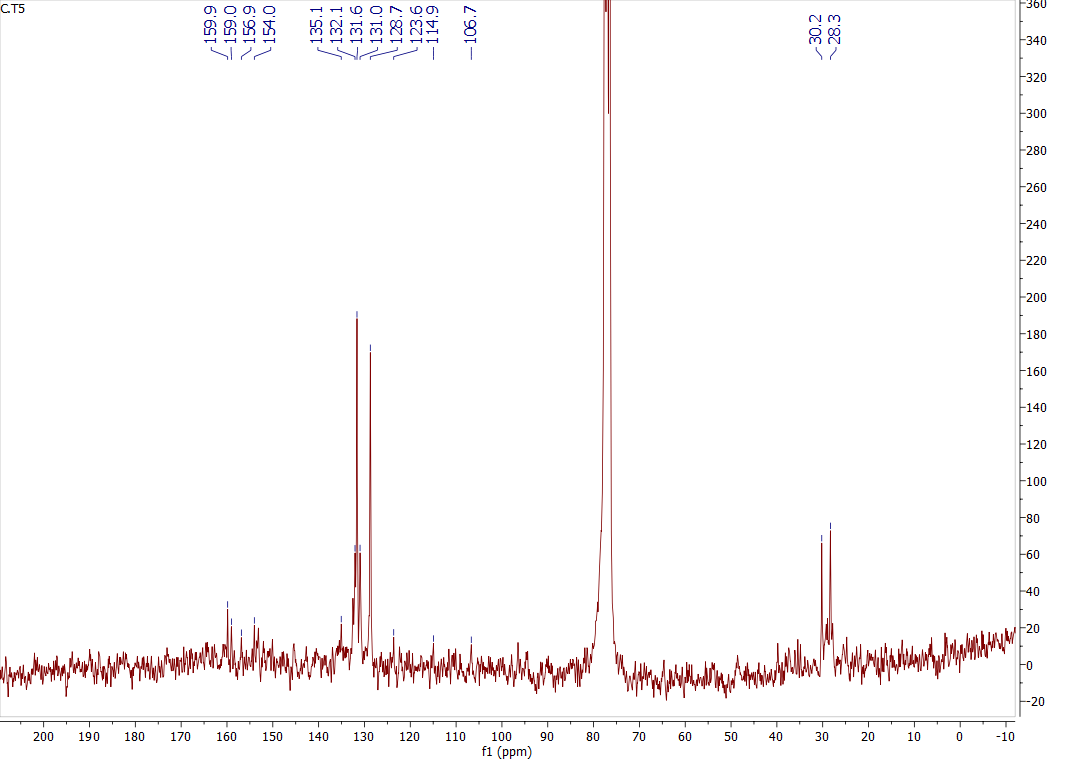


*Figure S18. Spectrum ^13^C NMR (63 MHz) of 7-amino-5-(4-bromophenyl)-1,3-dimethyl-2,4-dioxo-1,2,3,4-tetrahydropyrido[2,3-d]pyrimidine-6-carbonitrile in CDCl_3_ solvent*

**7-amino-1,3-dimethyl-5-(4-nitrophenyl)-2,4-dioxo-1,2,3,4-tetrahydropyrido[2,3-d]pyrimidine-6-carbonitrile (4h)**


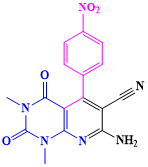


Melting point= >300 °C,

FT-IR (KBr, ν cm^-1^): 3456, 3335, 3110, 2214, 1694, 1603, 1514, 1442, 1346, 1151, 1108, 857, 790, 505.


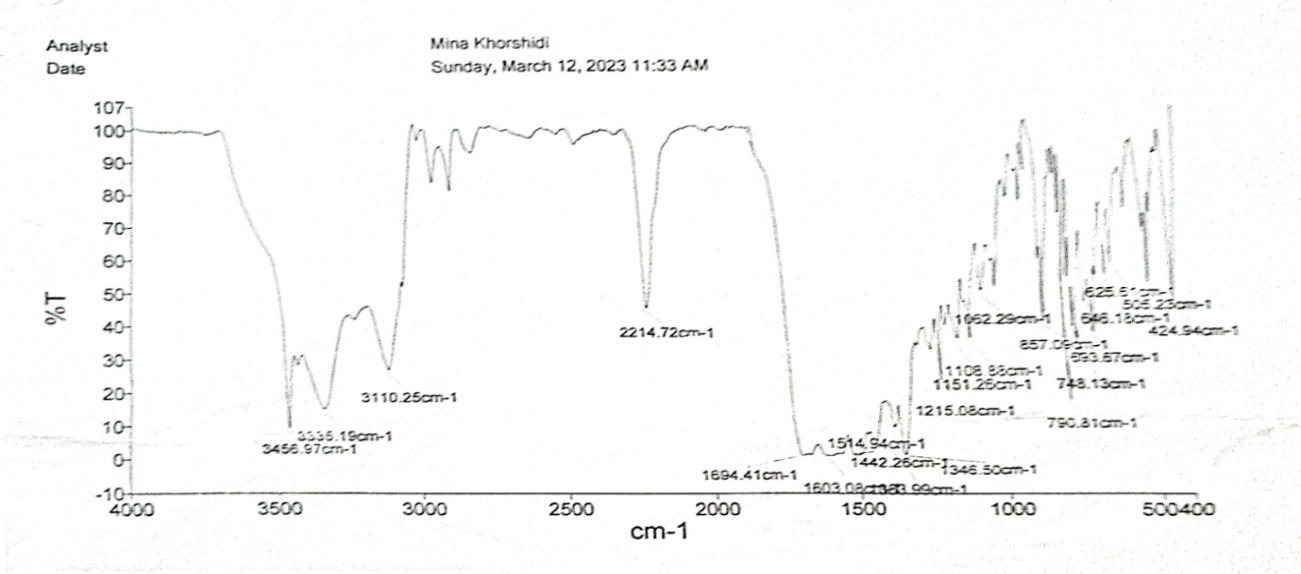


*Figure S19. Spectrum FTIR of* *7-amino-1,3-dimethyl-5-(4-nitrophenyl)-2,4-dioxo-1,2,3,4-tetrahydropyrido[2,3-d]pyrimidine-6-carbonitrile*
